# Laos’ Social Health Insurance (SHI) program’s impact on older people’s accessibility and financial security against catastrophic health expense

**DOI:** 10.1186/s12913-023-10063-z

**Published:** 2023-11-29

**Authors:** Somdeth Bodhisane, Sathirakorn Pongpanich

**Affiliations:** College of Public Health Sciences (CPHS), Chulalongkorn University, Bangkok, Thailand

**Keywords:** Healthcare financing, Catastrophic health expenditure, Health service research, Older population

## Abstract

Laos has introduced various SHI schemes for multiple groups of the population, such as government officials and other population groups under the NHI schemes. There is no specific health insurance policy for this group of people who need special health services and may have a higher possibility of entering financial catastrophe. This study aims to assess the impact of SHI schemes on accessibility and financial catastrophe against catastrophic health expenditures for older people in Laos. A structured questionnaire has been used to retrieve information from 400 older people across 39 villages in Kaysone Phomvihane District, Savannakhet province, the largest province in Laos. In the analytical process, this study used a cross-sectional study design and binary logistic regression models to predict the likelihood of accessing health facilities and experiencing financial catastrophe. The study outcome shows that the increase in age, occupation, number of older people within a household, and presence of chronic conditions increase the likelihood of using health services. Despite the existence of various SHI schemes, this study found that 74 out of 165 households reported using health services experienced catastrophic health expenditure. Several characteristics are associated with catastrophic health expenditure: age, income level, and gender are prone to suffer from catastrophic health expenditure. The difficult problems stem from the absence of comprehensive legislation regarding the older population. Recommendations for policymakers in various timeframes have been made, which cover short- and long-term policy proposals, including providing a specialized lane or fast-track for an older population, building health facilities exclusively for older people, and providing transportation services for older individuals living alone.

**Supplementary Information:**

The online version contains supplementary material available at 10.1186/s12913-023-10063-z.

## Background

Majority of lower/middle income countries, out-of-pocket (OOP) expenses are the primary source of healthcare financing. OOP refers to the cash payments made by the patient or family to the healthcare provider [1]. In an effort to strengthen the mobilization of healthcare resources, a number of lower/middle income countries have recently launched ambitious health reforms. Their goals are to provide their populations with universal healthcare coverage and financial protection against high healthcare expenditures and poverty. Protecting individuals from financial catastrophe and destitution resulting from the utilization of health services is a priority for policymakers. WHO has proposed that OOP health expenditure be considered catastrophic when it exceeds or equals 40% of a household’s non-subsistence income, i.e., the income remaining after basic needs are met [2]. However, few low- and middle-income countries have attained universal coverage [3]. People in lower/middle income countries have less access to health services than those in industrialized nations, and the poor have even less access to healthcare inside their own countries. Although a lack of financial means may impede access, there is also an inverse causal relationship between poverty and access to health services. When individuals delay or disregard necessary medical care, their health deteriorates, resulting in income loss and increased medical costs [4–6].

In lower/middle income nations, inequalities that lead to impairments in health are pervasive, and the poor are especially susceptible [7]. The relationship between poverty and access to healthcare is part of a larger cycle in which poverty creates sickness, and illness keeps people in poverty [8]. Geographic accessibility exerts an important influence on healthcare access in low and middle-income countries. As a substantial access barrier, it has been demonstrated that travel time or distance to healthcare facilities is negatively correlated with the utilization of healthcare services. Good roads, a rarity in many impoverished regions of lower/middle income nations, are required not only for patients to reach health facilities, but also for the easy distribution of pharmaceuticals and other supplies to health facilities, prompt emergency referrals, and enhanced supervision of health professionals. Access to health treatment is further hampered by inadequate communication services. This challenge becomes more critical in rural areas where unfavorable weather disrupts communication. Remote health centers need more time and money for travel-related charges, impeding the capacity of the poor to receive medical care [9, 10].

As a lower/middle income country in the Association of Southeast Asian Nations (ASEAN), the current population of Laos is at approximately 7.5 million in 2022, which is equivalent to 0.09% of the world’s population. Laos has been experiencing a tremendous increase in its population. According to official demographic projections, between 2015 and 2030, there will be a population increase of between 1.71 and 1.91 million people, resulting in a total population of between 10.25 and 10.72 million by 2050. The rates of population growth for various age groups will vary considerably. While the working-age and senior populations are projected to increase by 10.6% and 10.1%, respectively, the number of school-aged children [5–14] is projected to decrease by 3.7%. Laos does not currently have a problem with an increasing portion of older population. The percentage of population aged over 60 years old in Laos expects to shift from 6.8% to 2020 to more than 15.7% in 2050 [11, 12].

Respect for the older people remains a cherished tradition in Lao society. Until the end of their lives, older individuals typically reside with their family, children, and grandchildren. Important roles of senior citizens in society’s social and religious events, as well as in local government. The Lao Government is always concerned with the health and living conditions of senior citizens and retirees. In 2001, the policy and the National Committee for Older Persons were formulated and approved with the assistance of numerous international Organizations such as Help Age International in Asia Pacific Region, UN in Laos, as well as local governance employees. In 2004, the Lao National Policy for the older people has been approved, focusing on: healthcare (treatment and recovery), protection and welfare, education and data and information provision, income enhancement, and encouraging the older people to participate in social activities by transferring knowledge to the young generation. However, there are number of challenges, which includes the lack of comprehensive legislation regarding older adults, and limited data on the older population, including health conditions, diseases, poverty and vulnerabilities. Currently, there is no mechanism at the subnational level to promote senior citizens. Even in large cities, there are only a few facilities and programs that encourage social activities for older people. Personnel-wise, there are insufficient social workers to provide services for older individuals who require assistance [13]. To date, there is no specific healthcare privilege for older citizens; most healthcare services in Laos are provided by the government, which is comprised of three administrative levels (central, provincial, and district) and four tiers of service providers (central, provincial, district, and health center). Additional community-based services are now being specified as part of the ongoing effort to build a service delivery package. The Ministry of Health (MOH) is centrally responsible for the management and organization of health services, health information, human health resources, health funding, health sector development and planning, and international health cooperation. In addition to its regulatory responsibilities (food and drug safety, administration of drugs and equipment, and licensing of private health institutions), the MOH operates medical colleges and universities, national medical centers, and central hospitals.

Social Health Insurance (SHI) expenses account for a modest fraction of total expenses. In response to the issues of limited access to health treatments and lack of financial protection for the poor and vulnerable, a number of social health protection systems have been created in recent years. In 2016, just 4.4% of the General Government Health Expenditure was allocated to SHI, with the rest coming through formal sector programs. The proportion of SHI expenses is projected to increase in the upcoming years due to the government’s recent decision to start the National Health Insurance (NHI) plan in 2016, which merged these different social health protection systems and expand their coverage to the entire nation in 2018. By merging these three initiatives, the NHI targets the entire population of the informal sector, through the Health Equity Fund (HEF), the Community-Based Health Insurance (CBHI), and the Free Maternal Neonatal and Child Health (FMNCH) program. Despite a significant decline of out-of-pocket (OOP) expenditures in the proportion of the total health expenditure from over 60% in 2000, to barely 45% in 2016, OOP payments remain the nation’s primary source of funding for health. High reliance on OOP expenses causes considerable financial barriers to receiving healthcare treatments, and renders the poor more vulnerable to health shocks [14].

SHI offers both Outpatient Department (OPD) and Inpatient Department (IPD) services, with contribution rates varying based on the location of the provided health service. OPD patients are required to pay a flat rate of 5,000 LAK (0.35 USD) at a village health center, 10,000 LAK (0.7 USD) at a referral hospital, and 15,000 LAK (1 USD) at a provincial hospital. IPD services are only available in district hospitals and referral/provincial hospitals, and patients are required to pay a flat fee of 30,000 LAK (2 USD). Patients who are transferred from the OPD to the Intensive Care Unit or ICU are required to pay an additional 20,000 (1.35 USD) LAK in district hospitals and 15,000 LAK (1 USD) in referral/provincial hospitals. However, patients must also pay 25% (as a co-payment) for surgery or treatment that exceeds 5 million LAK (350 USD) [15–17].

Access to public health services may be difficult for older people due to their deteriorating health, diminished physical and social mobility, and limited financial resources [18]. Despite their increased health risks, many older people do not have access to sufficient quantities and quality of healthcare. Approximately 63% of respondents in a survey conducted in multiple nations reported that it was difficult to obtain medical care when needed. Affordability is a challenge for older individuals. Occasionally, older adults retain lowly jobs, rely on assets or family support to support themselves, or receive inadequate pensions. In regions where healthcare is not uniformly available and inexpensive, many older citizens forego preventive care and even treatment or pay for medical expenses at the expense of other necessities. When older individuals seek medical care, particularly in lower/middle income countries, they typically encounter medical professionals who are inexperienced with their particular health issues and medical services that are not adapted to their needs [19]. According to the United Nations Principles for Older Persons Adopted by General Assembly resolution, older people should have access to health care to maintain or regain optimal physical, mental, and emotional health, and to prevent or delay the onset of disease. Besides, older people should be able to get the right level of care in an institution, which includes protection, rehabilitation, and social and mental stimulation in a safe and humane environment [20]. Although the older population is not covering a large group of people in Laos, however, accessibility to health service utilization and catastrophic health expenditure of the older population have never been adequately handled. With the introduction of SHI, this study aims to find the impact of SHI on accessibility and financial catastrophic against catastrophic health expenditure for older people in Laos.

## Methods

### Geographical scopes of study

It should be recalled that before implementing the NHI at the national level, the NHI was piloted in several provinces, including Savannakhet Province. As the capital of Savannakhet Province, Kaysone Phomvihane District was purposively selected as the study site [21]. Kaysone Phomvihane district is one of the fifteen districts with an annual per capita income of US$2,041. Kaysone Phomvihane has two hospitals: the Savannakhet Provincial Hospital (the main hospital) and Kaysone Phomvihane District Hospital. Over the Mekong River, the Second Thai–Lao Friendship Bridge connects the Mukdahan Province of Thailand to the Kaysone Phomvihane District. With a population of 125,760, Savannakhet province is the second-largest city in Laos after Vientiane Capital [10].

### Study design

A cross-sectional study design is utilized to predict the likelihood of accessing health facilities and experiencing financial catastrophe as a result of healthcare consumption of the older population in Kaysone Phomvihane District, Savannakhet Province, Laos. The Andersen Behavioral model is a framework used in health services research to understand and explain the factors that influence an individual’s utilization of healthcare services. It was developed in the 1960s and has been widely utilized to analyze healthcare utilization patterns across populations and settings. This model comprises predisposing, enabling, and need-based characteristics; these characteristics have been used to identify variables that may affect accessibility and catastrophic health expenditures [22]. Specifically, predisposing characteristics describes the likelihood of using health care services. Individuals are more or less likely to use health services based on their age, gender, and position in society. Enabling characteristics contain resources found within the family and community. The economic status and location of a family are examples of family assets. Access to healthcare facilities and the availability of aiding individuals are included among the community’s resources. Lastly, need-based characteristics cover the perception of demand for health services [22].

### Sample size calculation and data collection techniques

This sampling technique, n = z^2^ [p(1-p)]/ e^2^ = [(1.96)^2^ × (0.5 × 0.5)] / (0.05)^2^ = 384 [23]. Based on the 2022 census report, there are 67 villages in the Kaysone Phomvihane District, subdivided into 13 village groups throughout the province. This study randomly chose 39 villages, or three villages from each village group. As the leader of the data collection team, the corresponding author systematically selected ten respondents from various households where senior citizens were present, resulting in 390 respondents in total. Additional ten respondents were randomly selected from various villages. Hence, the total number of sample size was 400 participants.

In terms of data collection, the research team systematically selected households with at least one senior citizen from the Village Head’s list. Despite the fact that the life expectancy in Laos is at 68 years, the retirement age in Laos is usually 60 years [24]. This research categorizes the older population into three groups: 60–69 years old, 70–79 years old, and more than 80 years old [25]. The inclusion criteria include households with a family member aged more than or equal to 60 years old, whereas the exclusion criteria are households that have been living in the surveyed district for less than 6 months. Heads of households are expected to answer the question on behalf of the households’ senior members. All information retrieved from respondents was traced back up to 12 months (1 year). The data collection was conducted in March-May 2022 after the lockdown policy from Covid-19 was relieved. A Structured questionnaire was developed for this study based on the lesson learned from the previous study [21]. The data collection team was led by the corresponding author, with the assistance of 4 nurses from Savannakhet Provincial Hospital.

### Statistical techniques

This research uses both descriptive and inferential statistical methods to achieve its objectives. There are two logistic regression models. The binary logistic regression Model 1 is used to estimate the likelihood of local hospital admission. Hospitalization is the proxy for accessibly to health service utilization. Model 2 examines the likelihood of suffering catastrophic health expenditures in a local hospital.

In the binary logistic regression Model 1, hospital admission has been identified as the dependent variable, whereas independent variables are under Andersen’s Behavioral Model, namely, (1) redisposing characteristics include “age”, “level of education”, “household size”, and “occupation”, (2) Enabling characteristics are “income level”, “means of transportation”, “travel time to a hospital”, and (3) Need-based characteristics compose - the presence of older persons and chronic conditions within a household. Recall that OOP medical expenses are considered catastrophic when they reach or exceed 40% of a household’s non-subsistence income. On the other hand, the possibility of having a catastrophic health expenditure is the dependent variable for Model 2. In this model, independent variables are identical to the variables in the model 1.

### Validity

With the assistance of an expert from the College of Public Health Sciences at Chulalongkorn University, Thailand, the content validity of the instrument (structured questionnaire) was systematically evaluated to ensure that it had all the necessary data. In addition, construct validity was employed to confirm that the theoretical foundation supports the conceptual framework, which comprises many sources of information, interviews with key informants, and the establishment of a chain of evidence [26]. Typically, construct validity is determined by comparing the test to other tests that measure similar qualities to determine the degree of correlation between the two measures. Assessing construct validity is particularly important when investigating something that cannot be directly measured or observed [27, 28].

## Results

As mentioned in the data collection part, this study collects information from 400 respondents. According to Table 1, this study examined different measurements of older citizens and their household characteristics associated with hospital admission. Those characteristics include older individuals’ gender, age, level of education, household size, occupation, transportation method, traveling time to hospitals, presence of older individual(s), and presence of chronic condition(s) within their households. At first glance, it is observable that the Pearson Chi-Square of all the aforementioned variables is statistically significant at a 95% confidence interval, which technically means that the relationship between the sub-variables and hospital admission was not independent. A 95% confidence interval is a range of values that contains the true population mean with 95% assurance [29].

**Table 1:** Older people’s and their households’ characteristics associated with hospital admission in one-year period

Older people’s and their households’ characteristics	Hospital admission			Pearson X^2^
	No	Yes	Total	
**Predisposing characteristics** Gender				
♣ Male ♣ Female	196 (83.4%) 39 (16.6%)	31 (18.8%) 134 (81.2%)	227 (56.8%) 173 (43.3%)	0.01
Age				
♣ Young old (60–69) ♣ Middle-old (70–79) ♣ Senior (more than 80)	106 (45.1%) 81 (34.5%) 48 (20.4%)	53 (32.1%) 46 (27.9%) 66 (40%)	159 (39.8%) 127 (31.8%) 114 (28.5%)	0.01
Level of education				
♣ No formal education ♣ Primary school ♣ Secondary school ♣ College/ university degree	12 (5.1%) 25 (10.6%) 18 (7.7%) 180 (76.6%)	8 (4.8%) 56 (33.9%) 74 (44.8%) 27 (16.4%)	20 (5%) 81 (20.3%) 92 (23.0%) 207 (51.7%)	0.01
Household size				
♣ Small (1–4 people) ♣ Large (more than 5 people)	156 (66.4%) 76 (33.6%)	27 (16.4%) 138 (83.6%)	183 (45.8%) 217 (54.3%)	0.01
Occupation				
♣ Retired government official ♣ Self-employed or business owner ♣ Freelancer ♣ Others	74 (31.5%) 88 (37.4%) 56 (23.8%) 17 (7.2%)	30 (18.2%) 49 (29.7%) 23 (13.9%) 63 (38.2%)	104 (26%) 137 (34.3%) 79 (19.8%) 80 (20.0%)	0.01
**Enabling characteristics** Income level				
♣ Less than or equal to $125 ♣ More than $125 to $280 ♣ More than $280 to $500 ♣ More than $500	39 (16.6%) 76 (32.3%) 58 (24.7%) 62 (26.4%)	61 (37%) 61 (37%) 19 (11.5%) 24 (14.5%)	100 (25%) 137 (34.3%) 77 (19.3%) 86 (21.5%)	0.01
Means of transportation				
♣ Without a vehicle ♣ Motorcycle ♣ Car	16 (6.8%) 103 (43.8%) 116 (49.4%)	36 (21.8%) 76 (46.1%) 53 (32.1%)	52 (13%) 179 (44.8%) 169 (42.3%)	0.01
Travelling time to hospital				
♣ Less than 30 minutes ♣ Between 30 to 60 minutes	165 (70.2%) 70 (29.8%)	92 (55.8%) 73 (44.2%)	257 (64.3%) 143 (35.8%)	0.02
**Need-based characteristics** Presence of elderly populations				
♣ One ♣ Two or more	183 (77.9%) 52 (22.1%)	66 (40.0%) 99 (60%)	249 (62.3%) 151 (37.8%)	0.01
Presence of chronic conditions within households				
♣ No ♣ Yes	218 (92.8%) 17 (7.2%)	45 (27.3%) 120 (72.7%)	263 (65.8%) 137 (34.3%)	0.01
Total	235	165	400	

According to Table 1, it can be seen that households reported having hospital admission within a one-year period are primarily in income quartile 1 (less than or equal to $125) and quartile 2 (more than $125 to $280). Hospital admission from these two quartiles covers over 122 or 74% of total hospital admission reports of 165 counts. This finding is related to the fact that most of the households visiting hospital do not have any means of transportation (within their households) or have a motorcycle, which covers 21.8% and 46.1%, respectively. In terms of time travel to the hospital, the majority of the patients, 55.8%, can access health service utilization within 30 min. The values of Pearson X^2^ of all older people’s and their households’ characteristics are statistically significant at 95% CI, notifying that the relationships between these variables and hospital admission were not independent.

**Table 2:** Older people’s and their households’ characteristics associated with catastrophic health expenditures due to health service utilization in one-year period

Older people’s and their households’ characteristics	Catastrophic health expenditure			Pearson X^2^
	No	Yes	Total	
**Predisposing characteristics** Gender				
♣ Male ♣ Female	10 (11%) 81 (89%)	21 (28.4%) 53 (71.6%)	31 (18.8%) 134 (81.2%)	0.04
Age				
♣ Young old (60–69) ♣ Middle-old (70–79) ♣ Senior (more than 80)	25 (27.5%) 23 (25.3%) 43 (47.3%)	28 (37.8%) 23 (31.1%) 23 (31.1%)	53 (32.1%) 46 (27.9%) 66 (40%)	0.104
Level of education				
♣ No formal education ♣ Primary school ♣ Secondary school ♣ College/ university degree	7 (7.7%) 36 (39.6%) 39 (42.9%) 9 (9.9%)	1 (1.4%) 20 (27%) 35 (47.3%) 18 (24.3%)	8 (4.8%) 56 (33.9%) 74 (44.8%) 27 (16.4%)	0.014
Household size				
♣ Small (1–4 people) ♣ Large (more than 5 people)	9 (9.9%) 82 (90.1%)	18 (24.3%) 56 (75.7%)	27 (16.4%) 138 (83.6%)	0.011
Occupation				
♣ Retired government official ♣ Self-employed or business owner ♣ Freelancer ♣ Others	14 (15.4%) 33 (36.3%) 14 (15.4%) 30 (33%)	16 (21.6%) 16 (21.6%) 9 (12.2%) 33 (44.6%)	30 (18.2%) 49 (29.7%) 23 (13.9%) 63 (38.2%)	0.135
**Enabling characteristics** Income level				
♣ Less than or equal to $125 ♣ More than $125 to $280 ♣ More than $280 to $500 ♣ More than $500	19 (20.9%) 40 (44%) 12 (13.2%) 20 (22%)	42 (56.8%) 21 (28.4%) 7 (9.5%) 4 (5.4%)	61 (37%) 61 (37%) 19 (11.5%) 24 (14.5%)	0.01
Means of transportation				
♣ Without a vehicle ♣ Motorcycle ♣ Car	10 (11%) 45 (49.5%) 36 (39.6%)	26 (35.1%) 31 (41.9%) 17 (23%)	36 (21.8%) 76 (46.1%) 53 (32.1%)	0.01
Travelling time to hospital				
♣ Less than 30 minutes ♣ Between 30 to 60 minutes	52 (57.1%) 39 (42.9%)	40 (54.1%) 34 (45.9%)	92 (55.8%) 73 (44.2%)	0.405
**Need-based characteristics** Presence of elderly populations				
♣ One ♣ Two or more	36 (39.6%) 55 (60.4%)	30 (40.5%) 44 (59.5%)	66 (40%) 99 (60%)	0.512
Presence of chronic conditions within households				
♣ No ♣ Yes	26 (28.6%) 65 (71.4%)	19 (25.7%) 55 (74.3%)	45 (27.3%) 120 (72.7%)	0.406
Total	91	74	165	

According to Table 2, cross-tabulation statistics between respondent/ households’ characteristics and catastrophic health expenditures for the older group due to IPD service. The case study survey of 400 respondents it can find out that 165 respondents report using IPD or health service utilization within the previous year. According to the comparison between household income and health-related expenditure, it shows that there 74 households (approximately 44.85%) encounter catastrophic health expenditure.

Besides, the descriptive analysis also found that most of the patients from two lower income quartiles (“less than or equal to $125” and “more than $ 125 to $ 280”) share the high portion of encountering catastrophic health expenditure more than 85.2%, of which, 56.8% falls into the poorest. The presence of chronic conditions within households seems to be an important factor leading to catastrophic health expenditures. In particular, a household with at least one-member suffering from chronic health issues covered 74.3% when compared to that 25.7% of the household without any chronic health condition. Under “level of education”, respondents who graduated with a secondary school degree have the highest degree of catastrophic health expenditure of 47.3%, which is higher in comparison to respondents with “no formal education,” “primary school,” and “college and university degree.”

**Table 3:** Accessibility of health service utilization in 1-year period

Older population’s and their households’ characteristics (Independent variables)	Binary logistic regression model 1: Accessibility to health service utilization (in 1-year period)	
	Nagelkerke R^2^ = 0.745	
	Odd Ratio (OR)	P-Value
**Predisposing characteristics** Gender		
♣ Male		
♣ Female	3.966	0.127
Age		
♣ Young old (60–69)		
♣ Middle-old (70–79) ♣ Oldest-old (more than 80)	4.393 6.396	0.03* 0.01*
Level of education		
♣ No formal education		
♣ Primary school ♣ Secondary school ♣ College/ university degree	0.394 0.867 0.739	0.464 0.919 0.853
Household size		
♣ Small (1–4 people)		
♣ Large (more than 5 people)	4.186	0.092
Occupation		
♣ Retired government official		
♣ Self-employed or business owner ♣ Freelancer ♣ Others	3.348 0.639 19	0.061 0.643 0.01*
**Enabling characteristics** Income level		
♣ Less than or equal to $125		
♣ More than $125 to $280 ♣ More than $280 to $500 ♣ More than $500	1.705 2.690 4.541	0.446 0.296 0.128
Means of transportation		
♣ Without a vehicle		
♣ Motorcycle ♣ Car	0.361 0.401	0.383 0.490
Travelling time to hospital		
♣ Less than 30 minutes		
♣ Between 30 to 60 minutes	0.957	0.925
**Need-based characteristics** Presence of older persons		
♣ One		
♣ Two or more	29.702	0.001*
Presence of chronic conditions		
♣ No		
♣ Yes	14.363	0.001*

The binary logistic regression model 1 is used to predict the accessibility to health service utilization of the older population. The Nagelkerke R^2^ of this model is approximately 0.745, which seems to be very high. It should be noted that the Nagelkerke R^2^ has a range of values between 0 and 1. It measures how much of the total variation of the dependent variable can be accounted for by the independent variables in the current model. From Table 3, the model shows that several independent variables significantly impact health service utilization (IPD services), which are age, occupation, presence of the older people, and chronic conditions within households.

The binary logistic regression model 1 shows that the higher the age of respondents, the higher the probability of using a health service utilization within one year. The model indicates that the oldest-old group (over 80 years old) has approximately 6.930 times higher chance of using IPD health services than the young-old (60–69 years old) at a 95% confidence interval. The middle-old group (70–79 years old) has a relatively lower rate of 4.393 times higher chance than the young-old group at a 95% confidence interval. Under occupation, the “others”^1^ group has a significantly higher possibility of using health service utilization 19 times than a retired government official at a 95% confidence interval. Regardless of not being statistically significant at 95%, “self-employed or business owners” have approximately 3.348 higher odds of using a hospital when compared to the retired government official group. Under Andersen’s need-based Characteristics, a household with two or more older persons is predicted to have a 29.702 times higher probability of using IPD service than a household with only one senior citizen at a 95% confidence interval. Households with the “presence of chronic conditions” are 14.363 times more likely to use health service utilizations when compared to households without any chronic conditions at a 95% confidence interval. Last but not least, despite not being statistically significant at a 95% confidence interval, it is observed that higher-income households (of more than $500) are approximately 4.541 times more likely to use access to health services utilization.

**Table 4:** Possibility of encountering catastrophic health expenditures due to health Service utilization in 1-year period

Older population’s and their household’s characteristics (Independent variables)	Binary logistic regression model 2: Possibility of encountering catastrophic health expenditure (in 1-year period)	
	Nagelkerke R^2^ = 0.434	
	Odd Ratio (OR)	P-Value
**Predisposing characteristics** Gender		
♣ Male		
♣ Female	0.048	0.045*
Age		
♣ Young old (60–69) ♣ Middle-old (70–79) ♣ Oldest-old (more than 80)	0.714 0.254	0.570 0.011*
Level of education		
♣ No formal education ♣ Primary school ♣ Secondary school ♣ College/ university degree	12.021 16.570 30.359	0.308 0.206 0.131
Household size		
♣ Small (1–4 people)		
♣ Large (more than 5 people)	0.242	0.528
Occupation		
♣ Retired government official		
♣ Self-employed or business owner ♣ Freelancer ♣ Others	0.533 2.778 0.344	0.363 0.530 0.265
**Enabling characteristics** Income level		
♣ Less than or equal to $125		
♣ More than $125 to $280 ♣ More than $280 to $500 ♣ More than $500	0.183 0.028 0.012	0.014* 0.018* 0.002*
Mean of transportation		
♣ Without a vehicle		
♣ Motorcycle ♣ Car	0.401 0.813	0.219 0.859
Travelling time to hospital		
♣ Less than 30 minutes		
♣ Between 30 to 60 minutes	0.718	0.513
**Need-based characteristics** Presence of older persons		
♣ One		
♣ Two or more	0.242	0.528
Presence of chronic conditions		
♣ No		
♣ Yes	0.466	0.345

As mentioned in the descriptive statistics, there are 165 households that reported using IPD services within the one-year period. In this regard, the binary logistic regression model 2 uses this information to predict the probability of households with an older group of the population experiencing catastrophic health expenditure. Based on Table 4, the regression model shows that gender, age, and income level are statistically significant at a 95% confidence interval.

Under predisposing characteristics, female respondents seem to have a lower probability of encountering catastrophic health expenditure, about 20.83 times (invert OR – 1/0.048 compared to male respondents) at a 95% confidence interval. Furthermore, the oldest-old group of respondents has approximately 3.94 times (invert OR – 1/ 0.245) lower probability of facing financial catastrophe at a 95% confidence interval. Andersen’s enabling characteristics also play a critical role; in particular, a household with income “more than $500” has around 83.3 times (invert OR – 1/ 0.012) lower probability in comparison to the poorest group, which makes a living of “less than or equal to $125” at a 95% confidence interval. In other words, it can be said that the poorest income quartile has the highest possibility of experiencing catastrophic health expenditure. Despite not being statistically significant at a 95% confidence interval, another essential point from the findings is that older persons with college/ university degrees were more likely to face catastrophic health expenditure when compared to other groups.

## Discussions

Similar to other Southeast Asia countries, the Laos’ public pension system is weak, with insufficient benefits and coverage for the older population. Difficulties in obtaining healthcare-related services result in unmet healthcare needs, delayed care, and inadequate management of chronic illnesses, leading to increased health service utilization and the possibility of encountering catastrophic health expenditures [30]. The logistic regression model 1 has observed that the oldest-old (more than 80 years old) and middle-old (70–79 years old) group of older citizens have a higher probability of using healthcare utilization compared to the young-old group (60–69 years old). Since the probability of using health services has been a proxy for accessibility to health service utilization, it means that SHI enables health service utilization for the oldest group of people. Due to deteriorating health, those above 85 years old are far more likely to live in a long-term care facility than younger older adults. In fact, persons over the age of 85 years old are four times more likely to live in a nursing facility than those aged 75 to 84 years old [31]. A study in Norway found that the use of other healthcare services is high among middle-aged and older individuals but declines among the oldest old. Utilization of care services, particularly institutional care, increases with age [32]. Despite not reaching the 95% confident interval, it is able to realize that richer people are more likely to use health services. This could be because they are more affordable in non-medical related expenditure, which cover opportunity costs of followers, transportations, VIP room services and many other not cover under SHI [21]. Under predisposing characteristics, respondents’ occupation also plays an important role in accessibility to health service utilization. It can be seen that “other” occupations significantly had a higher chance of accessing health service utilization. These groups include farmers, housewives, senior citizens not in the labor force, and others that live with their working children. As a big family and availability of assistance, these people could easily access health services. A household with multiple senior citizens is more likely to utilize health services than a household with only one senior citizen. This finding does not come as a surprise, given that the greater the number of senior citizens, the greater the likelihood of hospital admission as well. Older patients utilize hospitals more often than younger patients; they have more emergency department admissions, longer hospital stays, and consume more hospital resources [33]. The good part is that this finding also indicates that the SHI makes it easier for households with a large number of older members to utilize health services when necessary. Moreover, despite not being statistically significant at a 95% confidence interval, large households were 4.186 more likely to use health service utilization when compared to smaller households of 4 members. In other words, these people do not have difficulty accessing health services when needed. In the developed counties’ contexts, after adjusting for patient demographic and clinical variables, older patients living alone were 50% more likely to end up needing the emergency department [34]. It is clear from this that older people in Laos still require special assistance from family members to access healthcare services. Compared to older people in developed countries who can use public emergency services and have timely access to health care, Lao’s older people who do not live with their children or other family members find it challenging to access healthcare. This means that regardless of the fact that older patients live alone, the government still plays an important role in providing special assistance to health service utilization. Another important finding found that the existence of chronic conditions significantly plays a very powerful role in health service utilization. The presence of the older population in the household and chronic conditions relate with an increased likelihood of utilizing health services in both domestic and international contexts. This statement is related to a study in China, which shows that the older population are more likely to suffer from at least one of four chronic diseases, including diabetes mellitus, hypertension, chronic obstructive pulmonary disease, and stroke [34].

The second binary logistic regression model found that four factors were statistically significant, namely: gender, age, and income level. Firstly, a household with the oldest female household member was more likely to suffer catastrophic health expenditure. This outcome could be supported by the fact that women may live longer than men on average, but this does not necessarily imply that they are healthier. Women over the age of 65 are at a higher risk than males for a number of ailments and are impacted differently by a number of diseases that affect both sexes. Heart disease, cancer, and cardiovascular diseases are the common causes of death for older women, just as they are for men. However, women are more frequently affected by various chronic illnesses, such as diabetes, hypertension, and arthritis [35]. Bangladesh, India, and South Korea provided evidence that households with chronically ill members were more likely to have catastrophic health expenditure [36–39].

Regardless of the no-service cost for most IPD services. Patients’ households can suffer from nonmedical expenditures, room services, food expenditures, medicine costs, and co-payment expenses. The lowest income quartile group has the highest possibility of experiencing catastrophic health expenditure. The result is in tandem with the previous study, which found that there is a negative correlation between income and the likelihood of incurring catastrophic health expenses. Poor households are prone to incur catastrophic health expenditures due to large nonmedical costs incurred during hospital admission, regardless of whether they are covered by NHI. These nonmedical expenditures may include travel expenses, food prices, room service fees, and other expenses [40]. Moreover, households that use public transportation always pay more than their counterparts, which proportionally increases the risk of incurring catastrophic medical costs. This study’s results are closely related to a case study conducted in the Republic of Korea, which demonstrates that despite the National Health Insurance system, a social insurance program that reduces the burden of OOP medical expenses, the amount of OOP expenses that individuals and their families are responsible for is still substantial, averaging 35.2% [41].

## Conclusions

There is a number of identifiable challenges, for instance: the lack of comprehensive legislation regarding older population. A subnational structure for the promotion of the older persons has not yet been devised. With the recent establishment of SHI, especially in remote areas, there are inadequate social workers and volunteers to provide services for older individuals in need of support. This is the first study ever conducted in Laos to assess the accessibility to health and catastrophic health expenditure for senior citizens in Laos. With the application of both descriptive and inferential statistics, it is possible to find out that the oldest-old (more than 80 years) do not have any issues with both access to health services and catastrophic health expenditure related to health services due to the existence of NHI. However, households categorized in the lowest income quartiles still have higher probability of encountering catastrophic health expenditure. Households with high medical expenses may be required to reduce other expenses. Due to the high risk of catastrophic health expenses, low-income households are more likely to spend their household income on health services under these unfavorable health conditions. This could lead to a lower standard of living, financial risks, and an increased probability of poverty.

### Policy recommendations

Regarding this study’s outcome, several policy recommendations have been proposed. Firstly, Lao health facilities still lack certain privilege policies that would facilitate the utilization of health services, such as a fast-track lane for senior citizens. As a result, older individuals must wait for many hours to receive proper medical care. In addition, the lack of designated parking areas for senior citizens during rush hours complicates the transportation process and may delay the timely utilization of health services. From this point, the government needs to facilitate access to health service utilization for the older citizens group, such as special parking spaces, a fast track to reducing waiting time, and reducing unnecessary documentation procedures. As part of Lao culture, senior family members are always followed by a younger family member to serve or even cook at the hospital. In this regard, to reduce the possibility of non-medical expenditures, it is necessary for the hospitals to serve (with fee or in the form of copayment) nutritious food for the older persons during their hospital admission. Moreover, for a household without any means of transportation, patients may have to take public transportation such as Tuk Tuk (auto rickshaw), taxi, serviced ambulance, and bus, which are costly and unformattable. The government may have to find solutions for senior citizens, such as free of charge or copayment of transportation services for older individuals to reduce this portion of non-medical expenditures. As the economy becomes more developed, the older population in larger cities is more likely to live alone than in smaller rural cities. As a result, it is recommended that the government develop a call center/emergency service pilot project in major cities solely for senior citizens. This service may include free consultation and (with copayment) consulting services for the older population. In addition, one of the government’s possible short- and medium-term responses is to develop health education and promotion campaigns for the older population. This project seeks to increase awareness of the older population in urban and rural areas. Efforts to improve basic knowledge and health awareness will indirectly reduce government funds subsidized for SHI and the older individuals’ quality of life. For the longer-term policy, at the provincial or regional hospitals, the government needs to build new buildings and facilities exclusively for senior citizens. This policy will effectively enhance better service and accessibility and reduce the waiting time for older persons. The outcomes of this study expect to illustrate accessibility to health services utilization and financial projects for the older population in Savannakhet Province and Laos. The result can also be generalized in other lower/middle income countries context that faces a dual burden from chronic communicable diseases and noncommunicable diseases.

### Limitations

Binary logistic regression models have been used to determine the effect of SHI on accessibility and financial catastrophe for Laos’ older population. Based on statistical analysis, several household characteristics are essential in accessing reliable health service utilization and encountering catastrophic expenditure. However, the possible limitation of this research is that this study doesn’t include information from in-depth interviews or focus group discussions, as it answers the “how” and “why” research questions and promotes a deeper comprehension of experiences, phenomena, and context [42]. This study was conducted in Savannakhet Province, the biggest province. In terms of generalizability, the outcome of this research could be comparable to other relatively better economic locations in Laos, such as Vientiane capital, Champasack, Luang Prabang, and other provinces. However, this study outcome does not focus on rural or poor areas of Laos, which could be regarded as another limitation of this study. Moreover, this study retrieved information within 12 months, but there is no mechanism to handle biased information during the data collection. From the limitation of this research, it is highly recommended that future studies should apply a qualitative-based study design that also focuses on the rural areas of Laos.

### Electronic supplementary material

Below is the link to the electronic supplementary material.

Supplementary Material 1

## Data Availability

Structured questionnaire and SPSS data based are available upon request to the corresponding author.

## Footnotes

1 farmers, housewives, senior citizens not in the labor force.
