# Supplementary material for: Laos’ Social Health Insurance (SHI) program’s impact on older people’s accessibility and financial security against catastrophic health expense
Source: BMC Health Serv Res. 2023 Nov 29;23:1317. doi: 10.1186/s12913-023-10063-z (PMC10688000; doi:10.1186/s12913-023-10063-z)
Supplement: Supplementary file 1 — Supplementary Material 1 [file 12913_2023_10063_MOESM1_ESM.docx]

**Questionnaire (unofficial translation)**

Questionnaire number ….

**General information**

1. Name of respondent --
2. Contact number --
3. Type of answer --  Your information;  Answer on behalf of household’s oldest person.
4. Village’s name --
5. Household’s total member --  Small (1-4 people);  Large (more than 5 people).
6. Presence of elderly population --  One;  More than one.

**Older person’s sociodemographic**

1. Gender --  Male  Female.
2. Age --  Young old (60-69);  Middle-old (70-79);  Senior (more than 80).
3. Level of education --  No formal education;  Primary school;  Secondary school;

College/ university degree.

1. Occupation --  Retired government official;  Self-employed or business owner;  Freelancer;

Others.

Socioeconomic status

1. Income level
   1. Monthly income range (Converted to USD) --  Less than or equal to $125;  More than $125 to $280;  More than $280 to $500;  More than $500.
   2. Monthly income in actual number                 USD
2. Means of transportation --  Without a vehicle (public transportation system);  Motorcycle;  Car.

Accessibility to hospital.

1. Travelling time to the closest hospital --  Less than 30 minutes;  Between 30 to 60 minutes.
2. Difficulty of accessing health care when needed --  Very easy;  Easy;  Average;  Difficult;

Very difficult.

1. Presence of chronic conditions within households --  No;  Yes.
2. Awareness about NHI/ SHI --  No;  Yes.
3. Health service utilization within 12 months --  No;  Yes (if yes please proceed to “Health service expenditure”

Health service expenditure

1. Monthly expenditure
   1. Monthly expenditure range (Converted to USD) --  Less than or equal to $125;  More than $125 to $280;  More than $280 to $500;  More than $500.
   2. Monthly expenditure in actual number                 USD.
2. Estimated total health expenditure related to IPD services (not covered by SH) --                 USD.
3. Estimated medical expenditure (not covered by SH) --                 USD.
4. Estimated nonmedical expenditure^[[1]](#footnote-1)^ --                 USD.

1. Non-medical expenditures are those that are essential to healthcare facilities but are not directly related to treatment. [↑](#footnote-ref-1)
